# Bitter and Sweet Diets Alter Taste Response and Alcohol Consumption Behavior in Mice

**DOI:** 10.3390/nu17050874

**Published:** 2025-02-28

**Authors:** Anna P. Koh, Robin Dando

**Affiliations:** Department of Food Science, Cornell University, Ithaca, NY 14853, USA

**Keywords:** taste, behavior, alcohol, diet

## Abstract

Background/Objectives: Taste guides the consumption of food and alcohol for both humans and rodents. Given that chronic dietary exposure to bitter and sweet foods are purported to alter the perception of bitter and sweet tastes respectively, we hypothesized that dietary habits may shape how the taste properties of ethanol are perceived and thus how it is consumed. Methods: Using C57BL/6 mice as a model, we contrasted taste behavior, morphology, and expression after a 4-week diet featuring consistent bitter, sweet, or neutral (water) stimuli. Results: Our results demonstrated that a 4-week bitter diet containing a quinine solution increased preference for ethanol, while a 4-week sweet diet consisting of a sucralose solution did not alter ethanol preference nor intake. The quinine diet also reduced the number of sweet- or umami-sensing T1R3-positive cells in the circumvallate papillae taste buds of the mice. Conclusions: Based on the behavioral changes observed with the bitter diet, it is possible that either bitter or sweet taste, or both together, drive the increase in ethanol preference. The implications of these findings for alcohol consumption are that dietary habits that do not necessarily concern alcohol may be capable of altering alcohol preference via taste habituation. Habitual intake of bitter and/or sweet foods can shift the perception of taste over time. Changes to how the taste components of alcohol are perceived may also alter how acceptable the taste of alcohol is when experienced as a whole, thereby having the unintended consequence of shifting alcohol consumption levels. Our study demonstrates another side to bitter habituation, which, thus far, has been studied in the more positive context of developing a set of dietary tactics for promoting bitter vegetable intake.

## 1. Introduction

### 1.1. Taste Guides the Consumption of Alcohol

Alcohol consumption is a confounding risk factor for many medical conditions, including three of the CDC’s top five leading causes of death in the United States: cancer, stroke, and unintentional injuries [1]. Over 14 million adults in the USA are afflicted with alcohol use disorder, which cost the U.S. USD 249 billion in 2010 alone [2]. To mitigate the disease burden of excessive drinking, extensive research has been conducted in search of the genetic basis for alcoholism. Variations in genes that encode GABAA receptor α-2 subunit (GABRA2), cholinergic muscarinic 2 receptor (CHRM2), and α-synuclein (SNCA) in the brain are a few loci that have been implicated as proffering a predisposition toward alcohol abuse [3,4,5]. Additionally, chronic drinking reinforces alcohol consumption behavior through modulation of neuroreceptors, such as the dopamine D1 receptor and serotonin receptor 1A (5-HT1A) [6,7]. Given that clinical treatment options often target specific receptors, understanding the physiological basis for alcoholism is instrumental in efforts to manage alcohol use disorder.

While alcohol consumption is widely influenced by neurobiological factors, its modulators are not confined to the brain. Research in the last two decades has expanded this narrative to also include findings concerning the taste system. Perceived as tasting both sweet and bitter in humans and rodents, alcohol is voluntarily consumed with varying degrees of acceptance [8,9,10] in a manner some have linked to taste [11]. In particular, functional polymorphisms in human bitter receptor gene TAS2R38—and to a lesser extent, TAS2R13 and TAS2R16—are associated with alcohol intake [12,13,14,15]. In a study that established bitter taste sensitivity as a reliable predictor for alcohol intake, Duffy et al. [12] demonstrated a positive correlation between perceived bitterness intensity of 6-n-propylthiouracil (PROP)—a bitter tastant and emblem for genetic variance in bitter perception, particularly that arising from common mutations in the bitter taste receptor TAS2R38—and bitterness from ethanol in human subjects. This result suggests that greater sensitivity to bitterness, which presumably leads to a higher degree of aversion to bitter taste, may curb alcohol intake.

At the other end of the spectrum, liking for sweet taste can drive alcohol intake [16,17,18,19]. Through a knockout experiment, Blednov et al. [18] showed that mice lacking taste signaling genes α-gustducin (GNAT3), transient receptor potential cation channel (TRPM5), or taste receptor gene T1R3 demonstrated lower preference for, and intake of ethanol compared to their wild-type counterparts. A positive correlation between preference for sweet taste and ethanol has also been observed in human subjects [20,21]. Additionally, in humans, ethanol is reported as having a burning sensation [22]. A physiological rationale for this phenomenon was verified in rodent studies showing that the heat-sensing vanilloid receptor-1 (TRPV1) mediates ethanol intake response in a manner similar to how humans and rodents sense spiciness from capsaicin [23,24], which is sensed independent of the taste buds.

Collectively, these results suggest that taste serves to guide not only our consumption of food but also alcohol. That both bitter and sweet tastes are specifically implicated in mediating alcohol intake was demonstrated in a study by Lanier et al. [11], which showed that PROP bitterness rating of scotch was inversely correlated with liking of its bitterness and sweetness in college students. Given the mounting evidence, it is fair to deduce that alcohol consumption patterns may be modulated by altering taste perception. Taste response in turn has been shown to readily change with habitual intake, which can lower perceived sensitivity to a habitually consumed stimulus [25,26,27,28]. For appetitive tastes such as sweet, umami, and moderate levels of salty, habitual intake reduces perceived intensity and raises the preferred dose of the respective tastant, which may reinforce a loop of unhealthy overconsumption [29,30]. Similarly, repeated exposure to aversive tastes such as bitter raises the tolerated intensity of the taste quality, which in turn allows for increased consumption of bitter tasting foods like cruciferous vegetables [31,32].

**Figure 1:**
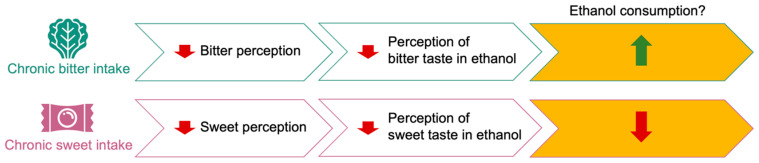
Central hypothesis, whereby consumption of a diet high in sweet or bitter tastants confers a weaker taste response, leading to increase (green arrow) or decrease (red arrow) in ethanol consumption.

Therefore, because the taste properties of ethanol—bitter and sweet—can be shaped by dietary habits, we hypothesized that diets rich in bitter or sweet tasting foods may also modulate the perception and consumption of ethanol. Specifically, blunted taste response as a result of chronic bitter or sweet diets may reduce the perception of the aversive bitter taste or appetitive sweet taste in ethanol, increasing or reducing ethanol consumption, respectively (Figure 1).

### 1.2. Repeated Exposure to Taste Stimuli Causes a Blunted Taste Response

Over the last five decades, a steady rise in food availability has introduced new diet-induced global health challenges [33]. In particular, excessive intake of highly palatable foods can lead to diets unhealthily high in sodium or sugars. Such diets are implicated in the development of chronic diseases such as diabetes and hypertension and have overtaken undernutrition as the number one cause of diet-induced death [34]. Burdens of disease tied to such diets are growing at an alarming rate, with Singh et al. [35] ascribing more than 180,000 mortalities in 2010 to sugar-sweetened-beverage-induced diabetes, cardiovascular diseases, and cancers based on a comparative risk assessment model.

Taste has been rated consistently as the most important factor influencing consumers’ purchasing decisions [36,37] and contributes vitally to feelings of satiation that affect meal cessation [38]. Further, overconsumption of highly palatable foods can induce changes in the perception of taste [30], thus altering the drivers of feeding behavior. Repeated exposure to taste stimuli can suppress taste responses, as in the case of habitual consumption of artificial sweeteners or monosodium glutamate producing a reduction in their perceived intensities in human subjects [39,40].

Diet-driven behavioral changes have been studied in the opposing context as well, in which subjects on a low-sugar diet reported heightened sensitivity to sweet taste [41,42]. Weighed in conjunction with a potential correlation between reduction in dietary sugar intake and a decrease in body weight [43], it is clear that taste-adaptation-induced changes in eating behavior should be considered as potential players in the management of diet-related health challenges.

### 1.3. The Taste System

There are five fully accepted basic taste modalities—sweet, salty, bitter, sour, and umami—detected by taste buds in the oral cavity and epiglottis, in both mice and humans. A taste bud is made up of 50–100 elongated specialized epithelial cells of three different sub-types: roughly 50% type I cells, 30% type II cells, and 20% type III cells [44]. 

Type I cells serve a glial-like function through their involvement in synaptic neurotransmission termination and ionic homeostasis maintenance [44,45] in addition to transducing salty taste through amiloride-sensitive epithelial sodium channels (ENaC) [46,47]. Type II cells sense bitter, sweet or umami stimuli through G protein-coupled receptors (GPCRs). Within type II cells, taste stimuli are sensed by the GPCR taste receptor type 1 (T1Rs) and taste receptor type 2 (T2Rs). Type II cells sense sweet, umami, and bitter tastes through heterodimeric T1R2–T1R3, T1R1–T1R3, and monomeric T2R receptors, respectively.

Expressed in type III cells, SNAP25 forms a SNARE complex with VAMP2 and Syntaxin 1A to mediate neurotransmitter release via exocytosis of synaptic vesicles and promote neural plasticity of taste nerves [48,49]. Given that P2X2–P2X3 receptors are essential for the transmission of neural signals from a taste stimulus to the brain via primary sensory afferents [50,51,52], it is conceivable that long-term dietary differences may affect taste innervation either in addition to or alternatively to taste receptor expression.

## 2. Materials and Methods

### 2.1. Animals

**Figure 2:**
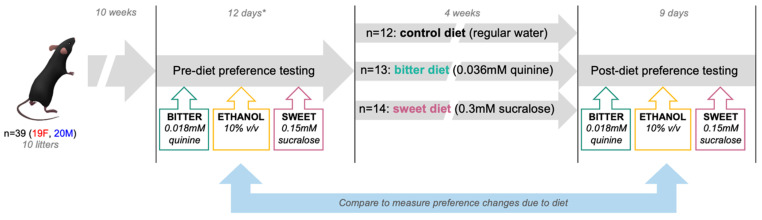
Schematic of study design showing pre-diet, diet, and post-diet periods, with 10 litters split into control, bitter, and sweet diet groups (n = 39). Two bottle preference tests of bitter, ethanol, and sweet solutions were performed pre- and post-diet. * Pre-diet preference testing included two consecutive rounds of bitter and sweet preference testing to eliminate potential learning effects.

Experiments were designed and performed in compliance with Cornell University’s Institutional Animal Care and Use Committee (IACUC) regulations. Animals used in this study were in-house bred, single-housed C57BL/6 male and female mice originally purchased from Jackson Labs (Bar Harbor, ME) maintained on an ad libitum diet of standard chow (Teklad 2918, global 18% protein rodent diet). At the start of the diet period, all mice were randomly assigned to a dietary treatment group by litter at 12 ± 1 weeks old. The diet period consisted of 4 weeks of treatment (Figure 2), in which animals would consume water with either no supplementation (control) or supplemented with quinine (bitter diet) or sucralose (sweet diet). In a pre-test period, mice weights were tracked to ensure no dehydration was occurring during the dietary treatment period—for example, from mice reluctant to drink quinine solutions—but as no weight loss was evident, body weights were not continuously tracked for the main study. As mice were treated with diets co-housed, data on individual consumption of dietary solutions in the 4-week period were not recorded.

### 2.2. Behavioral Testing

To test behavioral changes due to diet, mice were subsequently single-housed and underwent a series of two-bottle preference tests. Two-bottle preference testing is a method to determine how much a test solution is preferred over a control (usually water), providing mice with water and a tastant solution simultaneously for a prolonged period (48 h in our tests). Two 15 mL glass bottles were provided to each mouse, with spouts inserted into the cage side-by-side. Bottles were swapped in position at 24-h mark to minimize any side preference. Mice were given water only for 24 h in between each round of testing to offset potential carryover effects.

Diet-induced changes in ethanol preference and intake were measured by comparing pre- and post-diet two-bottle preference testing data. Weights of filled bottles were recorded at the start and end of each round of preference testing. The difference in pre- and post-testing weight was used to calculate ethanol preference and intake. Changes in bitter and sweet preference and intake were calculated as the primary outcome measure to enable comparisons with existing literature on the effects of diet on taste response and to delve into potential molecular mechanisms of taste habituation.

Prism 9.0.2 software (GraphPad, San Diego, CA, USA) was used to analyze and visualize behavioral testing results. Datasets were analyzed using a paired, parametric *t*-test if they passed the D’Agostino and Pearson normality test and a paired, non-parametric *t*-test if they did not.

### 2.3. Pre-Diet Preference Testing

Mice showed inconsistencies in the first round of two-bottle bitter preference testing in a pilot study, possibly due to neophobia of an aversive stimulus. Consecutive two-bottle bitter preference testing demonstrated a learning curve, in which mice collectively produced a more consistent range of data in the second round. Therefore, pre-diet preference testing began with an acclimatization round of bitter preference testing before the main test. Sweet preference testing was also repeated to eliminate a potential learning effect; however, no difference was observed for the appetitive stimulus. Therefore, the first round of sweet preference testing data was used in analysis to maintain the same comparison timeframe between pre- and post-diet.

### 2.4. Post-Diet Preference Testing

Testing sequence ran from the most aversive (quinine) to most appetitive (sucralose), with mildly appetitive ethanol in between, determined based on the recommendation that testing the most preferred tastant last will yield fewer carryover effects [53,54]. Following a four-week period in which the sweet diet group received 0.3 mM sucralose in drinking water, the bitter diet group received 0.036 mM quinine, and the control group consumed standard water, the same sequence and concentrations of testing were performed to compare pre- and post-diet preferences for quinine, ethanol and sucralose. A four-week period was deemed sufficient to see several rounds of cell turnover, as taste cells have an estimated half-life of 8–24 days [55,56], and changes in taste receptor expression have been reported on this timescale [57].

### 2.5. Tastant Concentrations

Concentrations of tastants used are summarized in Table 1. Quinine and sucralose concentrations for preference testing were chosen from a pilot study, in which sequential preference testing was performed with increasing concentrations of each tastant to assess the concentration at which appetitive tastant produced around 75% preference and aversive tastant produced around 25% preference; 10% ethanol was used to allow suitable comparisons to findings of previous studies. Diet concentrations were chosen at twice the preference testing concentration to produce a sizeable adaptation response within a physiologically relevant concentration range, while still ensuring that the bitter group would continue to consume water. A non-nutritive sweetener sucralose was selected as the sweet stimulus to minimize post-ingestive or metabolic effects of long-term diet treatment.

### 2.6. Immunofluorescence Staining

At the end of post-diet preference testing, mice were euthanized with CO_2_ followed by cervical dislocation, and tongues were collected. Circumvallate (CV) papillae were excised from tongue tissue and fixed in 4% PFA (Electron Microscopy Sciences, Hatfield, PA, USA), cryoprotected overnight in 30% sucrose (MilliporeSigma, Burlington, MA, USA), and frozen in OCT compound (ThermoFisher Scientific, Waltham, MA, USA). Then, 10-micron-thick coronal sections of the CV were cut using Thermo Scientific Microm HM 550 cryostat (ThermoFisher Scientific, Waltham, MA, USA) and placed on glass slides (Electron Microscopy Sciences, Hatfield, PA, USA).

A two-day protocol was followed to stain tissue sections for antigens listed in Supplemental Table S1. On the first day, slides were treated with 1% Triton X-100 (MilliporeSigma, Burlington, MA, USA), incubated with a blocking solution (3% BSA (Amresco, Solo, OH, USA); 3% Donkey serum (Equitech-Bio, Kerrville, TX, USA); 0.3% Triton X-100 (MilliporeSigma, Burlington, MA, USA)) at room temperature for 3 h, and then incubated with primary antibodies at 4 °C overnight. On the second day, slides were incubated with Alexa Fluor^®^-conjugated secondary antibodies (Invitrogen, Carlsbad, CA, USA) for 2 h and mounted with DAPI Fluoromount-G (Electron Microscopy Sciences, Hatfield, PA, USA) to visualize nuclei. Stained tissue slides were imaged with an Olympus IX71 microscope (Olympus Corporation, Tokyo, Japan) affixed to an ORCA-Flash 4.0 camera (Hamamatsu Photonics K.K., Hamamatsu City, Japan). Images were processed and analyzed using ImageJ v1.52 (NIH, Bethesda, MD, USA).

### 2.7. Quantification of Taste Buds and Taste Cells

Circumvallate papillae were sectioned across their entire coronal length, yielding between 35–50 slices per CV. There was no significant difference in the number of slices per CV in each group. Because each taste bud is roughly 50 microns in diameter [45], every fifth section (each measuring 10 microns) was stained to avoid double-counting of any taste bud [58]. To quantify differences in the number of bitter- and sweet-sensing cells pre- and post-diet, sections were co-stained for two type II cell markers, α-gustducin (GNAT3) and T1R3 (Supplemental Figure S2). α-gustducin is differentially expressed across the tongue: while it may be co-expressed with sweet receptors in fungiform papillae on the anterior portion of the tongue, it is predominantly co-expressed with bitter receptors in the CV in the posterior taste field, with little to no overlap with T1R3 receptors expressed in sweet- or umami-sensitive type II cells [59,60,61,62]. Thus, α-gustducin was used as a marker to quantify changes in bitter-sensing cells. Since the receptor for sweet stimuli is composed of the T1R2–T1R3 heterodimer, T1R3 was used as a marker to quantify sweet or umami-sensing cells. Taste buds per CV were counted from merged images of T1R3 and GNAT3 staining, which together highlight the core of taste cells.

### 2.8. Quantification of Taste Innervation

Taste innervation was quantified by measuring the intensity of P2X3. All images of CV sections were normalized against a standard for contrast and background, after which an auto-threshold was set for a randomly selected taste bud from each section. Then, highlighted pixels were summed to represent the number of nerve fibers in each taste bud. Since any highlighted pixel is counted towards intensity in ImageJ, normalized images of individual taste buds were used for analysis instead of entire CV sections to minimize the effects of staining artifacts.

### 2.9. mRNA Expression in Taste Buds

An additional group of mice on identical treatments was divided randomly into three groups, consuming a control (n = 10), bitter (n = 10), or sweet diet (n = 10). The sexes of the mice were divided evenly to explore the potential for sex-specific effects. To match the ages of the mice from behavioral testing, mice began diet treatment at 12 ± 1 weeks of age. Throughout the diet period, weight and water intake were monitored to ensure that treatment solutions did not significantly affect hydration.

At the end of the four-week diet period, mice were euthanized with CO_2_ followed by cervical dislocation, and tongues were collected. To preserve the integrity of their morphological structures, excised tongues were immersed in Normal Tyrode’s solution (135 mM NaCl, 5 mM KCl, 1 mM MgCl_2_, 2 mM CaCl_2_, 5 mM NaHCO_3_, 10 mM sodium pyruvate, 10 mM HEPES, 10 mM D-glucose; pH 7.4 ± 0.1) upon removal and kept submerged in calcium-free Tyrode’s solution (135 mM NaCl, 5 mM KCl, 10 mM sodium pyruvate, 10 mM HEPES, 10 mM D-glucose, 20 mM EGTA, 5 mM BAPTA; pH 7.4 ± 0.1) throughout the remainder of the protocol.

To isolate taste cells from the tongue, the circumvallate papillae, which contain the highest density of taste buds in the tongue, were injected sub-epithelially with an enzyme cocktail (2.5 mg/mL dispase type II, 1.0 mg/mL collagenase type I, 0.25 mg/mL elastase, 0.5 mg/mL DNase I). Following 20 min of incubation, the epithelium was peeled back to collect taste buds using a micropipette. After lysing the cells, RNA was extracted using the Absolutely RNA Nanoprep Kit (Agilent Technologies, Santa Clara, CA, USA), and then the reverse-transcribed cDNA was used to run qRT-PCR using a QuantStudio Flex Real-Time PCR System (Applied Biosystems, Waltham, MA, USA) to measure mRNA expression levels of genes encoding the following proteins in taste buds: β-actin (housekeeping); PLCβ2, GNAT3, and TRPM5 (taste transduction markers); SNAP25 and PGP9.5 (signal transmission markers); T1R2 and T1R3 (sweet taste receptors); T2R5, T2R8, T2R26, T2R37, T2R40, and T2R44 (quinine-activated bitter receptors); and T2R35, T2R38, and T2R39 (non-quinine-activated bitter receptors) (Supplemental Table S2). The expression level of each gene was quantified using three technical replicates, and the average of the three-cycle threshold (Ct) values was used to analyze relative changes in gene expression using the 2-ddCt method. Prism 9.0.2 software (GraphPad, San Diego, CA, USA) was used to analyze and visualize gene expression data.

## 3. Results

### 3.1. Bitter Diet Altered Ethanol Preference, as Well as Sweet and Bitter Preference

**Figure 3:**
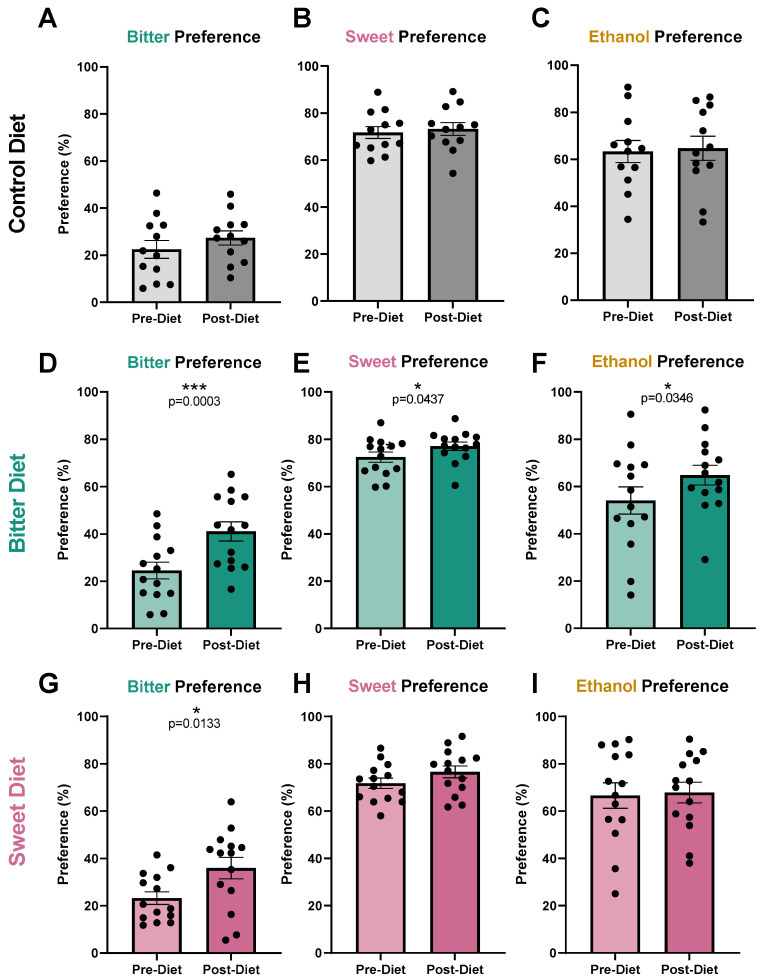
(**A**–**C**): Taste preference remained the same in the control group (mean with SEM, n = 12). Following a 4-week control diet (regular drinking water), there were no significant changes in preference for bitter, sweet, or ethanol. All datasets here and below passed the D’Agostino and Pearson normality test and were analyzed using paired, parametric *t*-test. (**D**–**F**): Bitter diet increased preferences for bitter, sweet, and ethanol (mean with SEM, n = 14). Following a 4-week bitter diet (0.036 mM quinine), bitter preference ((**A**); *p* = 0.0003), sweet preference ((**B**); *p* = 0.0437), and ethanol preference ((**C**); *p* = 0.0346) increased. (**G**–**I**): Sweet diet increased bitter preference (mean with SEM, n = 14). Following a 4-week sweet diet (0.3 mM sucralose), bitter preference increased ((**A**); *p* = 0.0133), with no change to sweet or ethanol preference. * denotes *p* < 0.05; *** *p* < 0.001; ns denotes not significant.

**Figure 4:**
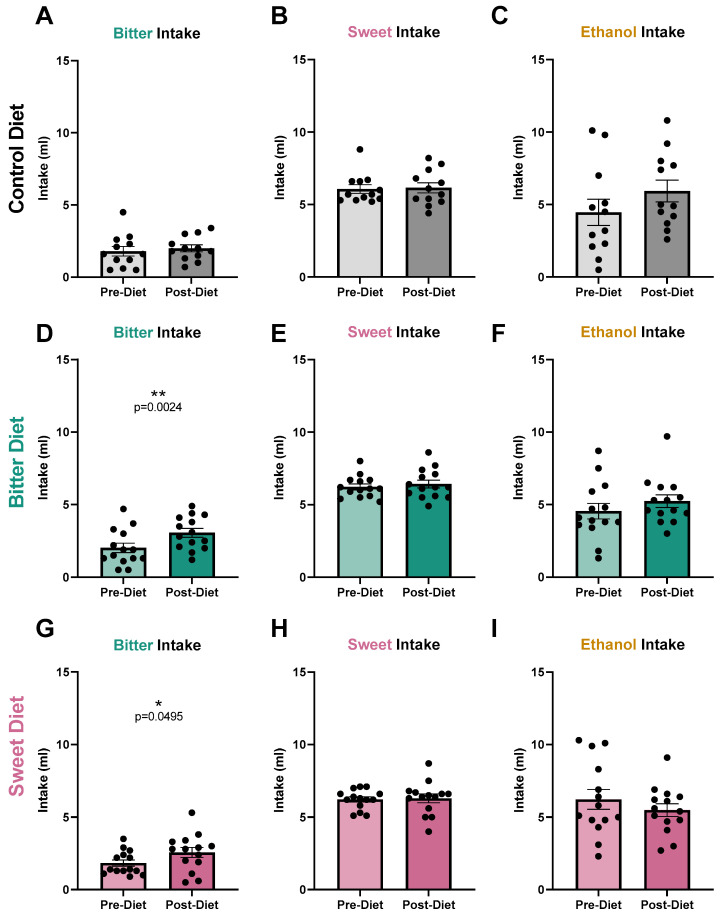
(**A**–**C**): Intake levels remained the same in control group (mean with SEM, n = 12). Following a 4-week control diet (regular drinking water), there were no significant changes in bitter, sweet, nor ethanol intake. All datasets except for sweet intake passed the D’Agostino and Pearson normality test and were analyzed using a paired parametric *t*-test. Sweet intake dataset was analyzed using paired, non-parametric *t*-test. (**D**–**F**): Bitter diet increased bitter intake (mean with SEM, n = 14). Following a 4-week bitter diet (0.036 mM quinine), bitter intake ((**D**); *p* = 0.0024) increased. All datasets except for ethanol intake passed the D’Agostino and Pearson normality test and were analyzed using paired, parametric *t*-test. Ethanol intake dataset was analyzed using paired, non-parametric *t*-test. (**G**–**I**): Sweet diet increased bitter intake (mean with SEM, n = 14). Following a 4-week sweet diet (0.3 mM sucralose), bitter intake ((**G**); *p* = 0.0495) increased. All datasets except for bitter intake passed the D’Agostino and Pearson normality test and were analyzed using paired, parametric *t*-test. Bitter intake dataset was analyzed using a paired, non-parametric test. * denotes *p* < 0.05; ** *p* < 0.01; ns denotes not significant.

Mice in the control group did not exhibit any changes in taste preference (Figure 3A–C) nor intake (Figure 4A–C) of bitter, ethanol, and sweet over the 4 week treatment period. Mice in the bitter diet group were supplemented with 0.036 mM quinine in drinking water for a total of 4 weeks. Compared to pre-treatment, bitter preference (Figure 3D; *p* = 0.0003) and intake levels measured post-treatment increased (Figure 4D; *p* = 0.0024), suggesting a blunting of bitter taste response, or an alteration in perceived hedonics for the stimulus, as a result of the 4-week bitter diet. Ethanol preference levels measured post-diet also increased in this group (Figure 3F; *p* = 0.0346), consistent with ethanol having a bitter taste, although intake levels did not change significantly (Figure 4F). Surprisingly, a significant increase in sweet preference was also observed in these mice (Figure 3E; *p* = 0.0437), which may have also contributed to the observed shift in ethanol preference. No change was observed in sweet intake (Figure 4E).

Mice in the sweet diet group were supplemented with 0.3 mM sucralose in drinking water over the course of 4 weeks. No change in sweet preference (Figure 3H, *p* = 0.1608) nor intake (Figure 4H, *p* = 0.8154) was observed. Preference and intake for ethanol also did not change (Figure 3I, *p* = 0.7836; Figure 4I, *p* = 0.3000). However, a sweet diet surprisingly led to a small increase in bitter preference (Figure 3G, *p* = 0.0133) and intake (Figure 4G, *p* = 0.0495) versus pre-treatment levels. No difference in the weights of mice from the control, bitter or sweet groups was observed throughout the diet period. Individual mouse behavioral data including before/after provided in Supplemental Table S3, Supplemental Figure S1.

### 3.2. Bitter Diet Reduced the Number of Sweet or Umami-Sensing Cells

**Figure 5:**
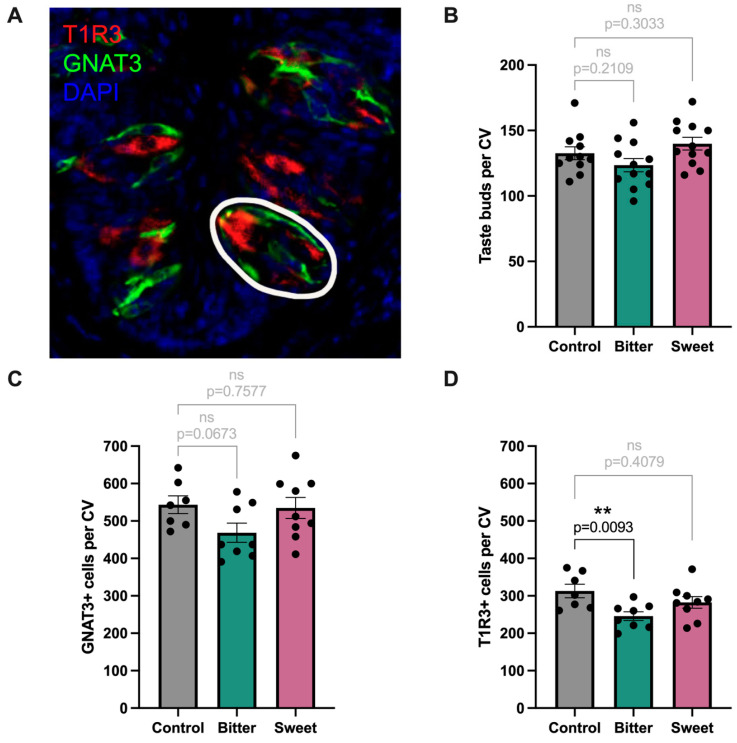
(**A**): Coronal sections of CV were stained and counted for bitter- (GNAT3; green) and sweet-sensing taste cells (T1R3; red) (control, n ≤ 11; bitter group, n ≤ 12; sweet group, n ≤ 12). (**B**): Taste buds, circled in A in white, were counted from merged images of (**C**): GNAT3+ and (**D**): T1R3+ staining. Bitter diet significantly reduced the number of sweet-sensing cells ((**D**), *p* = 0.0093).** denotes *p* < 0.01; ns denotes not significant.

CV sections were immunostained for the type II taste cell markers GNAT3 and T1R3 to detect changes in numbers of taste buds, bitter-sensing cells, and sweet or umami-sensing cells after a 4-week diet (Figure 5A). GNAT3 and T1R3 showed no colocalization, as confirmed by previous works [59,60,61,62], indicating that they serve as distinct markers for bitter- (GNAT3) and sweet- or umami (T1R3)-sensing cells. Since clusters of GNAT3+ and T1R3+ cells form taste buds, numbers of taste buds (Figure 5B), bitter-sensing cells (Figure 4C), and sweet- or umami-sensing cells (Figure 5D) were counted from these images. Neither diet affected the number of taste buds in the CV compared to the control (Figure 5B, bitter, *p* = 0.2109; sweet, *p* = 0.3033), nor did they affect the number of GNAT3 cells (Figure 5C, bitter, *p* = 0.0673; sweet, *p* = 0.7577). However, a comparison of bitter and control groups for T1R3 cell count showed a significant reduction in the number of sweet or umami-sensing cells associated with a bitter diet (Figure 5D, bitter, *p* = 0.0093), suggesting a potential bitter–sweet interaction from consumption of the bitter diet. No such effect was observed in the T1R3 cell count as a result of the sweet diet (Figure 5D, sweet, *p* = 0.4079). A further hypothesis may have been fewer type II cells on the whole in bitter-dieted animals, although this is speculative and was not specifically assayed here.

### 3.3. Neither Bitter nor Sweet Diet Altered Taste Innervation or Expression of Any Measured Genes in Taste Buds

CV sections were immunostained for P2X3 to detect changes in taste innervation after a 4-week diet (Supplemental Figure S3A). Average P2X3 intensity for a randomly selected taste bud from each CV section was quantified and showed no difference from the control in case of either the sweet or of the bitter diet (Supplemental Figure S3B: *p* = 0.8986 for bitter, *p* = 0.6296 for sweet).

Expression of five categories of taste-related genes were assayed: taste transduction, signal transmission, sweet receptors, quinine-activated bitter receptors, and non-quinine-activated bitter receptors. Markers for taste transduction—PLCβ2, GNAT3, and TRPM5—are involved in the type II taste cell signaling pathway and are discussed in the introduction (Supplemental Figure S4). Two markers of nerve fibers were included to assess potential changes in signal transmission: SNAP25 and PGP9.5 (Supplemental Figure S5). Genes encoding sweet receptors T1R2 and T1R3 are also discussed in the introduction (Supplemental Figure S6).

Unlike sweet taste, where the T1R2–T1R3 heterodimer is principally responsible for detecting all sweet stimuli, there are 34 bitter receptors in the mouse that vary in their receptive range in detecting bitter compounds [63]. Seven mouse bitter receptors known to respond to quinine were assayed: T2R5, T2R8, T2R15, T2R26, T2R37, T2R40, and T2R44 [63] (Supplemental Figure S7). To also test whether other bitter receptors change indirectly in response to quinine activation in nearby bitter cells, non-quinine-activated bitter receptors were also assayed: T2R35, T2R38, and T2R39 (Supplemental Figure S8).

One-way ANOVA did not reveal any significant differences between the groups for any gene, suggesting that behavioral changes observed were not due to changes on the mRNA level of genes assayed here. Expression levels of T2R15, T2R40, T2R44 were below the detection threshold for meaningful statistical comparisons to be made.

## 4. Discussion

### 4.1. Bitter Habituation Led to Increased Ethanol Consumption

A 4-week, 0.036 mM quinine diet increased ethanol preference in mice (Figure 3F). In addition to the confirmation of our hypothesis that a bitter diet would reduce the perception of the aversive bitter taste in ethanol and thus drive up its consumption, an increase in bitter preference and intake as well as an increase in sweet preference was observed. The biological importance of homeostatic maintenance and various opportunities for bitter-sweet taste interactions would suggest it is unlikely that the changes in perception of bitter, sweet, and ethanol are completely independent of one another; therefore, we cannot test correlation in these likely-dependent variables to establish causality. While we hypothesized that a bitter diet would act directly on bitter response to alter ethanol consumption, the unexpected behavioral change to sweet preference opens up additional potential avenues by which bitter diet alters ethanol preference. Based on the behavioral shifts observed, we can explore four potential scenarios by which chronic bitter intake may have increased ethanol preference: chronic bitter intake (1) blunts overall taste response to all stimuli, (2) enhances ethanol preference via altering bitter perception, (3) enhances ethanol preference via altering sweet perception, or (4) enhances ethanol preference via altering bitter and sweet perception.

In the first scenario, chronic bitter intake affects taste perception as a whole. It is possible that consistent stimulation of bitter receptors and their downstream signaling elements by 0.036 mM quinine—an aversive stimulus typically associated with ingestion of a dangerous substance—caused these animals’ taste systems to adapt to respond less over time once they learned to decouple the taste of quinine from the evolutionarily programmed signal to reject bitter substances for being potentially toxic. To further test this hypothesis, additional pre- and post-treatment preference tests to probe umami, salty, and sour tastes might help evaluate whether the blunting effect of a bitter diet extends across all taste modalities.

The second and third scenarios postulate that increased ethanol preference is driven by a bitter diet-induced change in either bitter or sweet perception. While both taste modalities have been linked to ethanol preference extensively, a surprising finding was that a bitter diet behaviorally altered sweet responses, which may in turn affect ethanol perception. To determine which taste modality is primarily linked to ethanol consumption behavior, Blednov et al. [18] bred KO mice lacking taste transduction genes Gnat3 and Trpm5 and the sweet or umami sensing receptor gene Tas1r3 and tested their preference for ethanol, saccharin, quinine, and NaCl. All three groups consumed less ethanol, and also exhibited reduced preference for saccharin, but not for quinine, demonstrating a relationship between the perception of sweet taste and ethanol. While the behavioral pattern we found was in the opposite direction, in that a reduction of T1R3+ cells was observed along with higher ethanol and sweet preferences, differences in test design and animal models mean we can only compare our results in terms of whether a trend exists. A reduction in T1R3+ cells in bitter-dieted mice suggests that this group’s ability to sense sweet and umami tastes may have been diminished; in turn, their blunted sweet taste may have led to an increased wanting of the sweet stimulus While we cannot establish causality based on our data, the evidence provided by the reduction of T1R3+ cells gives weight to the possibility that altered sweet response may have been a factor in the observed increase in ethanol preference.

Lastly, it is possible that, as in the study by Lanier et al. [11] that showed that both bitter and sweet tastes mediate scotch intake in college-aged students, the increased ethanol preference observed in our mice was driven simultaneously by changes in both bitter and sweet perception. Given that sucralose is known to activate bitter receptors [63], we should also consider the possibility that change in sweet response may in fact be an effect of the change in how mice are responding to the potential bitter taste of sucralose. Although our test sucralose concentration was below the detection threshold to activate bitter receptors, the lack of existing data on the effects of chronic bitter intake on perception of potentially bitter nonnutritive sweeteners means that we simply do not know how chronic bitter intake in our experiment may have affected how sucralose was perceived.

### 4.2. Bitter Habituation Is Behaviorally Documented, but Its Mechanism Remains Unclear

A 4-week bitter diet (0.036 mM quinine in drinking water) clearly reduced aversion to bitter taste in mice, measured as increases in quinine preference and intake (Figure 3 and Figure 4). This result was consistent with findings from Mura et al. [64] that 3 weeks of 0.03 mM quinine in drinking water was sufficient to produce an increase in quinine preference in female C57BL/6 mice. Moreover, Mura et al. confirmed that bitter habituation was not unique to quinine, showing similar reductions in aversion to other bitter stimuli (denatonium benzoate, caffeine, epigallocatechin gallate, L-tryptophan, L-isoleucine). In humans, bitter habituation has gained interest in the context of promoting healthy diets in children [31,32,65]. Broadly, results from human studies are aligned with those from rodent studies in that repeated exposure can increase liking and intake of bitter vegetables in children.

The collective data above establish clear evidence that a change in behavioral patterns occurs as a result of repeated exposure to a bitter diet. Regarding the molecular basis driving this behavioral change, several potential mechanisms have been postulated across various model organisms and will be discussed throughout this section, including receptor regulation [57], salivary protein regulation [66,67], and neural desensitization [68]. However, despite the relevance of bitter food consumption to human health, there is no consensus on the molecular mechanism underlying bitter habituation.

Previous work has hypothesized that receptor regulation may occur as an epigenetic response to repeated stimulation [57], similar to how chronic use of opioid agonists induces tolerance through µ-opioid receptor downregulation. Although T2R5 is the bitter receptor that is tuned to the widest range of agonists (activated by 45 out of 128 bitter substances in a heterologous expression system using HEK293T cells by Lossow et al. [63]), there are six other mouse bitter receptors also activated by quinine at threshold concentrations of 0.003–0.01 mM. The current work expands on the repertoire of both quinine-activated and non-activated bitter receptors, as well as receptors more broadly involved in taste transduction and signal transmission, in aiming to identify the molecular process by which bitter habituation occurs.

No changes in expression were detected in genes encoding PLCβ2, GNAT3, and TRPM5 (taste transduction markers); SNAP25 and PGP9.5 (signal transmission markers); T1R2 and T1R3 (sweet taste receptors); T2R5, T2R8, T2R26, and T2R37 (quinine-activated bitter receptors); or T2R35, T2R38, and T2R39 (non-quinine-activated bitter receptors), despite receptor mRNA levels being linked to taste response [69]. While these results do not provide a molecular explanation for behavioral effects of bitter habituation, they do allow us to rule out key bitter-taste-related genes as molecular drivers of this behavioral shift with diet under the given experimental conditions. Expression of the purinergic receptor P2X3 did not differ between bitter-dieted and control mice, indicating that the taste cell-sensory afferent junction remained unaffected at this particular level. However, this does not rule out the possibility that other elements of innervation could have been altered or that a different method of measuring P2X3 is necessary. In in vivo experiments with Manduca Sexta caterpillars, Glendinning et al. [68] demonstrated that an exposure-induced adaptation to caffeine leads to diminished firing rates of bitter-sensing cells. While the mechanism by which the firing rates of bitter-sensing cells were diminished is unclear, the results imply that a different method such as measuring neural responses in vivo may reveal changes that are not apparent in fixed tissues.

Finally, while saliva content was deemed outside the scope of this study, Martin et al. [66,67] proposed that bitter-diet-induced changes in salivary protein profiles may be responsible for increased bitter tolerance following chronic exposure. In previous reports [70,71], the group identified seven protein bands from saliva samples of male Long Evans rats that were altered by bitter (tannic acid and quinine) exposure. After a 2-week tannic acid diet, marker salivary proteins were upregulated, during which time increases in quinine intake and feeding rate were also observed, suggesting a potential link between an altered salivary protein profile and quinine tolerance [66].

### 4.3. Consequences of Sweet Diet on Taste May Be Confounded by Sucralose-Specific Effects

Despite evidence suggesting that chronic consumption of 0.15 mM sucralose should produce a sweet response that is altered sufficiently to affect how mice perceived the sweetness present in ethanol, responses to ethanol did not change (Figure 3I and Figure 4I), suggesting that consumption of a sweet diet at this level does not change ethanol consumption behavior. However, the sweet diet consisting of 0.15 mM sucralose also did not alter sweet response (Figure 3H and Figure 4H). Because our hypothesis that a sweet diet would alter ethanol perception was contingent on an altered sweet response, we could not reject our hypothesis solely based on the sweet habituation experiment described here. Additional data are necessary to determine whether the lack of change in ethanol perception is (1) sucralose-specific—for instance, by repeating the experiment with an iso-sweet concentration of sucrose to produce 60–80% preference—or (2) concentration-specific by repeating the experiment with a range of sucralose concentrations. In addition to the differences mentioned above between sucrose and sucralose, mechanistic differences may also be responsible for why a 4-week diet of 0.15 mM sucralose was insufficient to induce sweet habituation. Damak et al. [72] demonstrated in T1R3-KO mice that a loss of the sweet- or umami-sensing T1R3 receptor results in a near-loss in sensitivity to artificial sweeteners including sucralose, whereas sensitivity to sucrose was diminished, but remained at higher concentrations. Following this finding, researchers have shown that sucrose may produce sweet response through T1R3-independent pathways via pathways that depend on temperature or sensitivity to gurmarin, a selective T1R3 inhibitor [73,74], or that recruit sodium-glucose transporters [75]. That sucralose is unable to recruit these pathways may explain the discrepancy between our results and the altered sweet response reported with habitual sucrose intake and may further suggest that T1R3-independent pathways activated by sucrose are necessary in establishing sweet habituation.

What was notable about habitual sucralose intake was that it decreased aversion to bitter taste (Figure 3G and Figure 4G). We suspect this is a sucralose-specific effect, since there is an overlap in the bitter receptors activated by both sucralose and quinine (T2R5, T2R15, T2R44) and bitter response was measured by testing quinine preference. While sucralose purportedly activates these bitter receptors at higher concentrations than the 0.15 mM we used [63], the current work offers the possibility that chronic exposure to sucralose may have unforeseen effects on bitter sensitivity. Given the significance of daily sweetener use in the modern world, the effects of habitual sucralose intake on bitter taste perception may offer additional insights into how chronic consumption of beverages sweetened with non-nutritive sweeteners may alter our taste response.

### 4.4. Conclusions

The consumption of ethanol is not merely defined by its post-ingestive effects but can also be shaped by its sensory properties. In this study, we showed behavioral changes in taste due to 4-week-long bitter and sweet diets. Concurrently, a bitter diet led to an increase in ethanol preference alongside a change in bitter preference. On the other hand, a sweet diet did not change ethanol nor sweet preference but did alter bitter preference and intake, highlighting the possibility of bitter–sweet interactions and/or sucralose-specific effects of this sweet diet. From a molecular standpoint, a bitter diet caused a reduction in the number of sweet- or umami-sensing cells. Taken together, these results suggest that alterations in taste response from exposure to intensely tasting diets can influence the consumption of ethanol unrelated to a desire to consume alcohol for its effects on the body and highlights another potential health-related outcome influenced by diet.

## Figures and Tables

**Table 1:** Concentrations of tastants used in the study. All chemicals were purchased through Sigma-Aldrich (St. Louis, MO, USA).

Tastant	Chemical	Preference Testing	Diet
Bitter	Quinine hydrochloride dihydrate (Q1125-10G)	0.018 mM	0.036 mM
Sweet	Sucralose (69293-100G)	0.15 mM	0.3 mM
Ethanol	Ethanol (1009712500)	10% *v*/*v*	0

## Data Availability

Data available on request.

## Supplementary Materials

The following supporting information can be downloaded at: www.mdpi.com/article/10.3390/nu17050874/s1, Supplemental Table S1: Primary antibodies for immunofluorescence staining; Supplemental Table S2: Primer pair sequences for qRT-PCR; Supplemental Table S3: Cohen’s d (effect sizes) for preference and intake before/after diet; Supplemental Figure S1: Intake before and after dietary treatment for control (A–C), bitter (D–F), and sweet (G–I) diets; Supplemental Figure S2: CV section immunostained with GNAT3 (marker for bitter-sensing cells), T1R3 (marker for sweet- and umami-sensing cells), and DAPI (cell nuclei). Circled in white is a taste bud; Supplemental Figure S3: (A): Representative image. (B): 4-week bitter or sweet diet did not affect taste innervation, as measured through average P2X3 intensity per taste bud; Supplemental Figure S4: Expression of genes encoding receptors involved in taste transduction PLCβ2, GNAT3, and TRPM5 did not change in response to bitter nor sweet diet; Supplemental Figure S5: Expression of signal transmission genes SNAP25 and PGP9.5 did not change in response to bitter nor sweet diet; Supplemental Figure S6: Expression of sweet receptor genes T1R2 and T1R3 did not change in response to bitter nor sweet diet; Supplemental Figure S7: Expression of quinine-activated bitter receptor genes T2R5, T2R8, T2R26, and T2R37 did not change in response to bitter nor sweet diet; Supplemental Figure S8: Expression of bitter receptor genes not activated quinine, T2R35, T2R38, and T2R39, did not change in response to bitter nor sweet diet.
