# Supplementary material for: Bitter and Sweet Diets Alter Taste Response and Alcohol Consumption Behavior in Mice
Source: Nutrients. 2025 Feb 28;17(5):874. doi: 10.3390/nu17050874 (PMC11901823; doi:10.3390/nu17050874)
Supplement: Supplementary file 1 [file nutrients-17-00874-s001.zip › nutrients-3480165-supplementary rev r1.pdf]

Supplemental Materials

Supplemental Table S1. Primary antibodies for immunofluorescence staining.

Antigen	Host	Supplier	Catalog No.	Dilution
GNAT3	Goat	Aviva Systems Biology (San Diego, California)	OAEB00418	1:1000
GNAT3	Rabbit	Santa Cruz Biotechnology (Santa Cruz, California)	sc-395	1:1000
T1R3	Goat	Santa Cruz Biotechnology (Santa Cruz, California)	sc-22458	1:1000
P2X3	Rabbit	MilliporeSigma (Burlington, MA)	AB5895	1:500

Supplemental Table S2. Primer pair sequences for qRT-PCR (Lossow et al., 2016; Tomonari et al., 2012).

Gene Symbol	Protein	Forward Sequence (5'–3')	Reverse Sequence (5'–3')
Actb	β-actin	CACCCTGTGCTGCTCACC	GCACGATTTCCCTCTCAG
Plcβ2	PLCβ2	GAGCAAATCGCCAAGATGAT	CCTTGTCTGTGGTGACCTTG
Gnat3	GNAT3	GCAACCACCTCCATTGTTCT	AGAAGAGCCCACAGTCTTTGAG
Trpm5	TRPM5	GTCTGGAATCACAGGCCAAC	GTTGATGTGCCCCAAAAACT
Snap25	SNAP25	GGCAATAATCAGGATGGAGTAG	AGATTTAACCACTTCCCAGCA
Uchl1	PGP9.5	AGGGACAGGAAGTTAGCCCT	GGGACAGCTTCTCCGTTTCA
Tas1r2	T1R2	AAGCATCGCCTCCTACTCC	GGCTGGCAACTCTTAGAACAC
Tas1r3	T1R3	GAAGCATCCAGATGACTTCA	GGGAACAGAAGGACACTGAG
Tas2r105	T2R5	GAATCATAGAAACAGGACCTCG	CTTTACAAAGGCTTGCTTTAGC
Tas2r108	T2R8	TTCTGATTTCAGCCCTCACC	CCAAAAGCTGGTCCTGTTTC
Tas2r115	T2R15	AGAGAATGTGTGCTGTTCTACG	TCTCACGCTTGCACCAATAC
Tas2r126	T2R26	TGGTTGAAGTGGAGATTCCC	TGGTTTCCCCAAAAGAACAG
Tas2r135	T2R35	TCAGGTACTGGATGTGGCAG	CAGCAGCCCCTCTTTATCAC
Tas2r137	T2R37	GTCTCAGCATCACTCGGCTTT	GCAGGCGAGCTGAATAGCA
Tas2r138	T2R38	TTCTACTGCCTGAAAATAGCCAGTT	AACAACCACTCTAGAAGCTCTCCATT
Tas2r139	T2R39	ACACACCCTGAACATGAGAAACA	GGCCTGCATATGAGCCTCTATG
Tas2r140	T2R40	ATGAATGCTACTGTGAAGTG	CTAAGGACCTGGGAGTTC
Tas2r144	T2R44	ATGGCAATAATTACCACAAATTC	CTACCTTTTAAGGTAAAGATGAA

Supplemental Table S3. Cohen's d (effect sizes) for preference and intake before/after diet.

	Preference			Intake		
	Bitter	Sweet	Ethanol	Bitter	Sweet	Ethanol
**Diet**						
Control	0.414	0.168	0.083	0.197	0.075	0.495
Bitter	1.164	0.63	0.571	0.878	0.224	0.377
Sweet	0.914	0.549	0.07	0.675	0.084	0.344

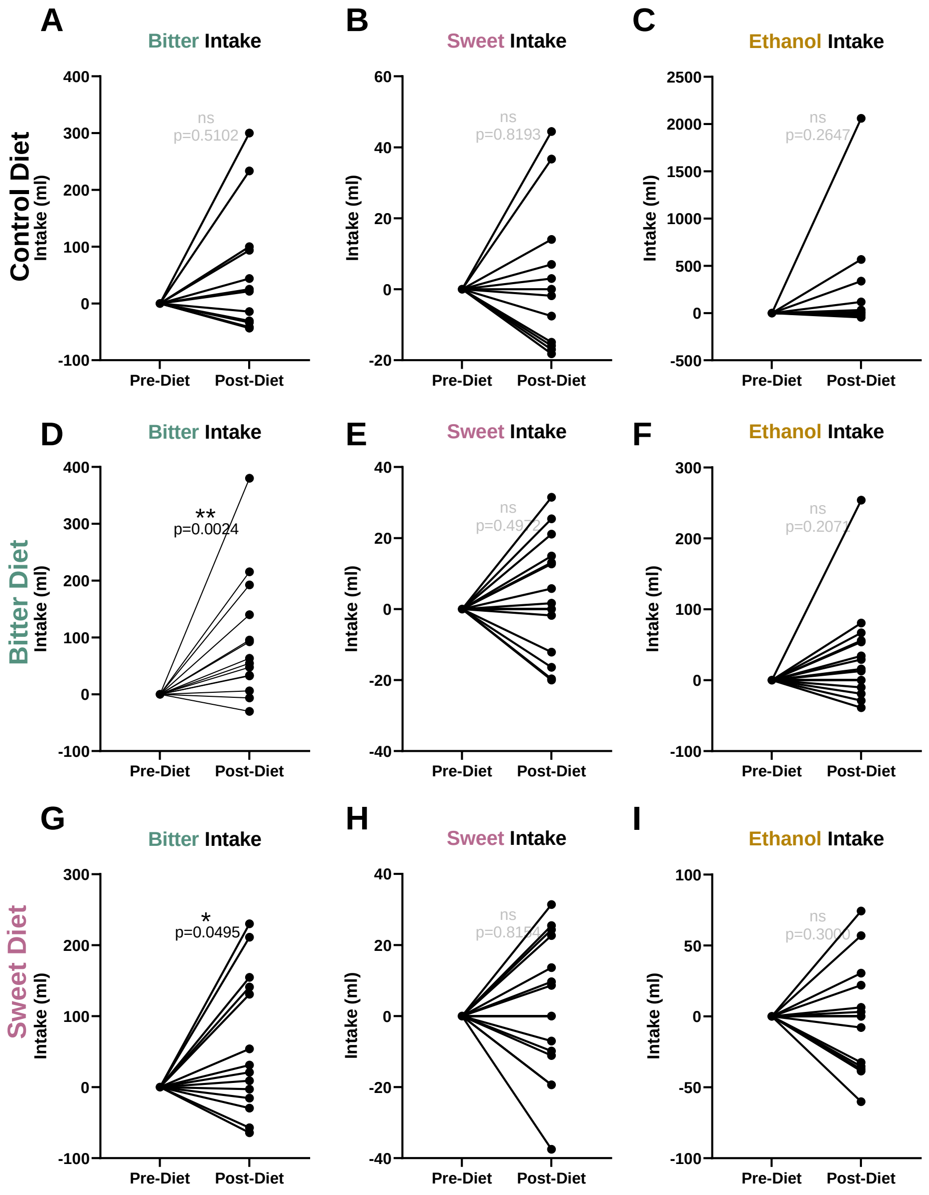

Supplemental Figure S1. Intake before and after dietary treatment for control (A-C), bitter (D-F), and sweet (G-I) diets.

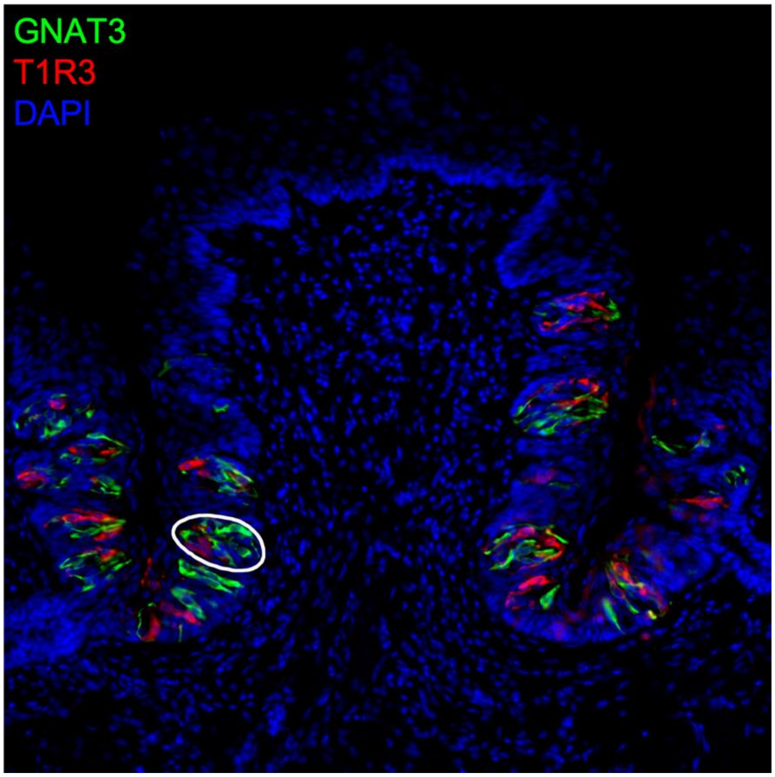

Supplemental Figure S2. CV section immunostained with GNAT3 (marker for bitter-sensing cells), T1R3 (marker for sweet- and umami-sensing cells), and DAPI (cell nuclei). Circled in white is a taste bud.

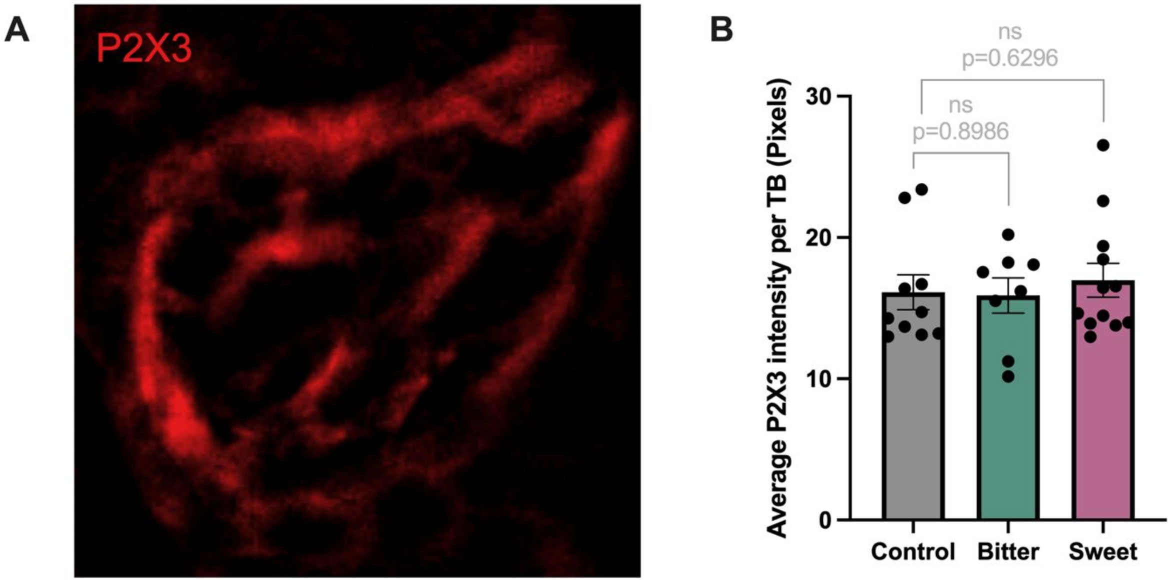

Supplemental Figure S3. A: Representative image. **B:** 4-week bitter or sweet diet did not affect taste innervation, as measured through average P2X3 intensity per taste bud.

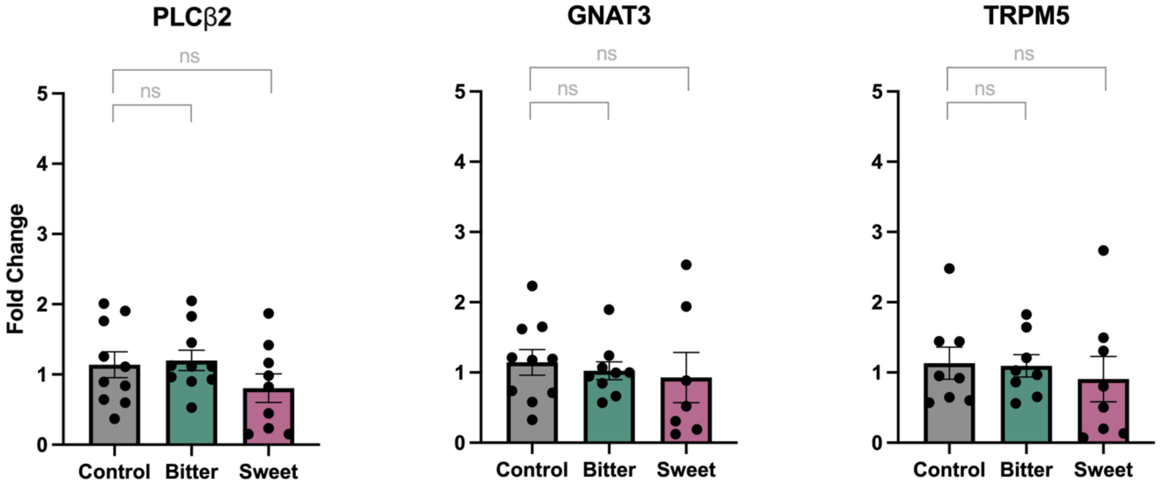

Supplemental Figure S4. Expression of genes encoding receptors involved in taste transduction PLCβ2, GNAT3, and TRPM5 did not change in response to bitter nor sweet diet.

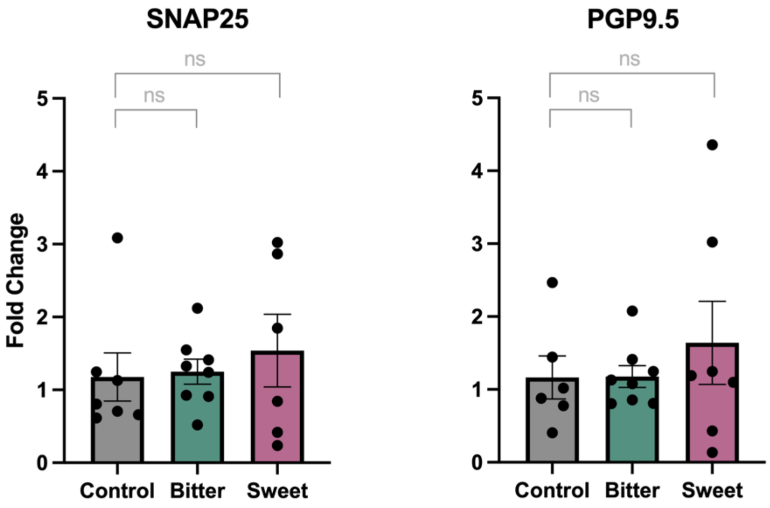

Supplemental Figure S5. Expression of signal transmission genes *SNAP25* and *PGP9.5* did not change in response to bitter nor sweet diet.

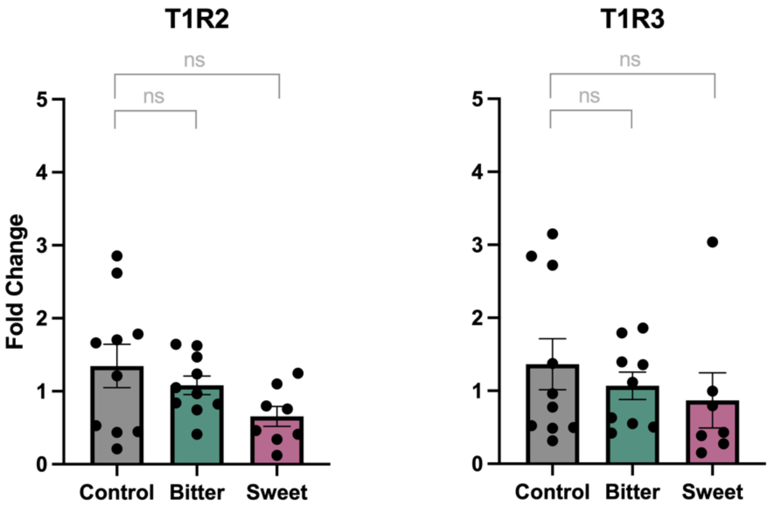

Supplemental Figure S6. Expression of sweet receptor genes *T1R2* and *T1R3* did not change in response to bitter nor sweet diet.

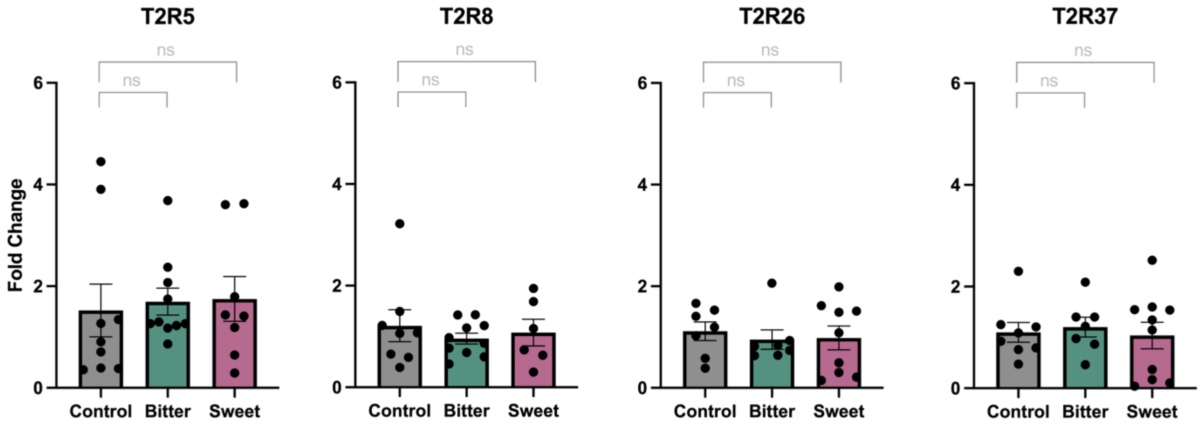

Supplemental Figure S7. Expression of quinine-activated bitter receptor genes *T2R5*, *T2R8*, *T2R26*, and *T2R37* did not change in response to bitter nor sweet diet.

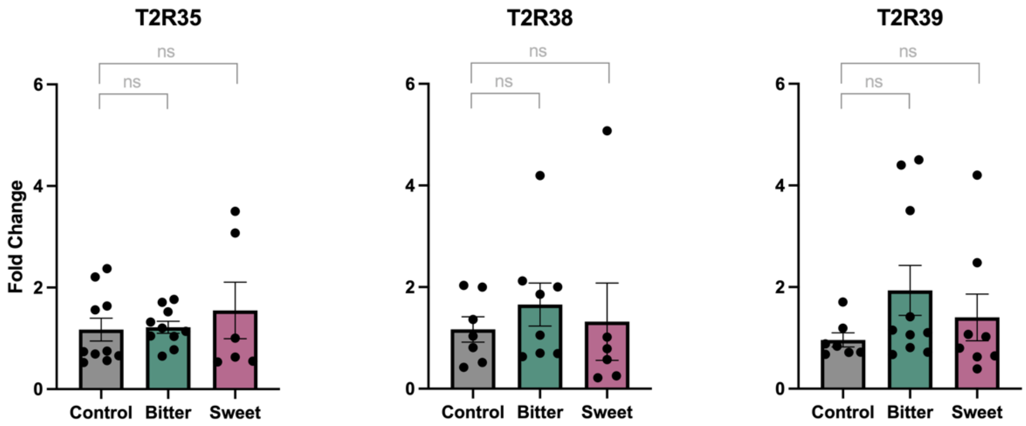

Supplemental Figure S8. Expression of bitter receptor genes *not* activated quinine, *T2R35*, *T2R38*, and *T2R39*, did not change in response to bitter nor sweet diet.
